# Human Umbilical Cord Blood Treatment in a Mouse Model of ALS: Optimization of Cell Dose

**DOI:** 10.1371/journal.pone.0002494

**Published:** 2008-06-25

**Authors:** Svitlana Garbuzova-Davis, Cyndy Davis Sanberg, Nicole Kuzmin-Nichols, Alison E. Willing, Carmelina Gemma, Paula C. Bickford, Christina Miller, Robert Rossi, Paul R. Sanberg

**Affiliations:** 1 Center of Excellence for Aging & Brain Repair, College of Medicine, University of South Florida, Tampa, Florida, United States of America; 2 Department of Neurosurgery, College of Medicine, University of South Florida, Tampa, Florida, United States of America; 3 Department of Molecular Pharmacology and Physiology, College of Medicine, University of South Florida, Tampa, Florida, United States of America; 4 Department of Pathology and Cell Biology, College of Medicine, University of South Florida, Tampa, Florida, United States of America; 5 Department of Psychiatry, College of Medicine, University of South Florida, Tampa, Florida, United States of America; 6 Saneron CCEL Therapeutics, Inc., Tampa, Florida, United States of America; University of Giessen, Germany

## Abstract

**Background:**

Amyotrophic Lateral Sclerosis (ALS) is a multicausal disease characterized by motor neuron degeneration in the spinal cord and brain. Cell therapy may be a promising new treatment for this devastating disorder. We recently showed that a single low dose (10^6^ cells) of mononuclear human umbilical cord blood (MNC hUCB) cells administered intravenously to G93A mice delayed symptom progression and modestly prolonged lifespan. The aim of this pre-clinical translation study is to optimize the dose of MNC hUCB cells to retard disease progression in G93A mice. Three different doses of MNC hUCB cells, 10×10^6^, 25×10^6^ and 50×10^6^, were administered intravenously into pre-symptomatic G93A mice. Motor function tests and various assays to determine cell effects were performed on these mice.

**Methodology/Principal Findings:**

Our results showed that a cell dose of 25×10^6^ cells significantly increased lifespan of mice by 20–25% and delayed disease progression by 15%. The most beneficial effect on decreasing pro-inflammatory cytokines in the brain and spinal cord was found in this group of mice. Human Th2 cytokines were found in plasma of mice receiving 25×10^6^ cells, although prevalent human Th1 cytokines were indicated in mice with 50×10^6^ cells. High response of splenic cells to mitogen (PHA) was indicated in mice receiving 25×10^6^ (mainly) and 10×10^6^ cells. Significantly increased lymphocytes and decreased neutrophils in the peripheral blood were found only in animals receiving 25×10^6^ cells. Stable reduction in microglia density in both cervical and lumbar spinal cords was also noted in mice administered with 25×10^6^ cells.

**Conclusions/Significance:**

These results demonstrate that treatment for ALS with an appropriate dose of MNC hUCB cells may provide a neuroprotective effect for motor neurons through active involvement of these cells in modulating the host immune inflammatory system response.

## Introduction

Amyotrophic lateral sclerosis (ALS) is a progressive neurodegenerative disorder characterized by a loss of brain and spinal cord motor neurons that clinically manifests as a progressive muscular weakness leading to paralysis and death of patients by respiratory failure within three to five years of diagnosis [1], [2]. Most cases of ALS are sporadic; the familial (FALS), or genetically linked, form of ALS represents only 5 to 10 percent of all cases. About 20% of FALS cases are the result of missense mutations in the Cu/Zn superoxide dismutase (SOD1) gene on chromosome 21 [3]. Since the first SOD1 missense mutations were reported, the number of known mutations has increased to more than 114 (see review, [4]). Treatments that are available for this disease lack the capacity to arrest disease progression or repair motor neurons.

Development of an effective treatment is complicated by the diffuse nature of motor neuron death; cell therapy may be a promising new treatment for ALS. Cell transplantation theories are divergent as to whether grafted cells replace dying neurons or provide a protective environment for neural survival. Motor neuron replacement through transplantation does not seem an effective treatment strategy for ALS. Even in animal studies using neural stem cell treatments, few cells become neurons [5], [6] and there is no evidence that “new” neurons re-innervate muscle. Bruijn [7] suggest that “stem cells engineered to secrete growth factors or other factors required for neuronal survival” or to “stimulate endogenous stem cells in the brain to generate new neurons” may be more feasible for ALS than neural replacement. Moreover, therapeutic strategies to retard disease progression “seem to be a more realistic clinical approach as compared with neuronal replacement” [8]. Thus, neuroprotection is more likely and does not rely on neural cell sources. This suggestion is supported by a recent study showing that implantation of Sertoli-enriched testicular cells, “nurse cells” with the ability to produce cytoprotective proteins, into the L4-L5 ventral horn of G93A mice had a significant neuroprotective benefit to vulnerable motor neurons by possible secretion of neurotrophic factors [9].

Although numerous hypotheses about the etiopathology of this multifactorial disease have been proposed (see review, [10]), increasing evidence suggests involvement of the immune/inflammatory system [11]–[15] in ALS pathogenesis. Modulation of immune/inflammatory effectors in ALS could have a protective effect for dying motor neurons. Human umbilical cord blood (hUCB) may be preferable to other cell sources such as bone marrow or neural stem cells. The hUCB cells are low in pathogenicity and immune immature. hUCB contains a heterogeneous cell population rich in hematopoietic progenitor/stem cells [16]–[20], non-hematopoietic cells that can develop into cells of various tissue lineages [21]–[24] as well as T cells, B cells and monocytes. Moreover, it has been shown that cord blood lymphocytes produce great amounts of the anti-inflammatory cytokine IL-10 [25]. This plasticity of hUCB cells opens new approaches for their application in treatment of various disorders including neurodegenerative diseases (see review, [26], [27]). Depending upon the pathological microenvironment into which the hUCB cells are introduced, neuroprotective and/or trophic effects of these cells appear more likely than neural replacement. In an animal model of stroke, for example, we showed that hUCB treatment reduces inflammation and provides neuroprotection [28], [29] by secretion of GDNF or other growth factors [30]. Our research group [31] and others [32], [33] have shown that a single intravenous (iv) administration of a mononuclear cell (MNC) fraction from hUCB (MNC hUCB) into pre-symptomatic G93A SOD1 mice delayed disease symptom progression and extended lifespan. We demonstrated that ten to twelve weeks after delivery of a small hUCB cell dose (10^6^), the cells were found widely distributed in the brain, spinal cord, and the peripheral organs (mainly, in the spleen) [31]. We suggest that hUCB cells may improve disease outcome through a peripheral immunological mechanism and may provide a neuroprotective effect for motor neurons through active involvement of these cells in modulating the host immune inflammatory system response.

However, the mechanisms underlying the beneficial effect of hUCB cells for repairing the diseased motor neurons in ALS are still unclear and the efficiency of these cells in treating ALS has yet to be determined. The aim of this pre-clinical translation study was to optimize the dose of hUCB cells to retard disease progression in the G93A mouse model of ALS. First, we compared the effect of intravenously transplanted MNC hUCB cells at three different doses on lifespan and amelioration of behavioral deficits in the G93A mice. Second, we investigated the dose response effect of administered hUCB cells upon inflammatory induces in G93A mice.

## Results

### Effect of MNC hUCB cell administration on disease progression

**Figure 1:**
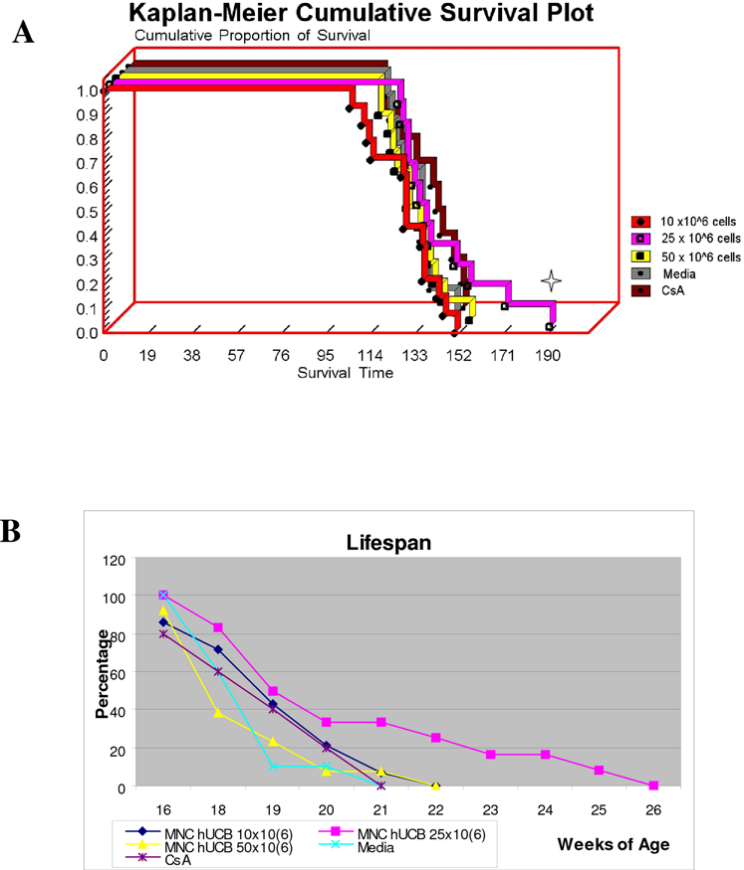
Effect of MNC hUCB cell administration on lifespan of G93A mice. A) Kaplan-Meier survival curves for G93A mice receiving 10×10^6^, 25×10^6^, and 50×10^6^ MNC hUCB cells. Control groups were Media and CsA. Significant (four pointed star) increase in survival between cell transplanted and Media-injected or CsA-injected groups was determined in mice receiving 25×10^6^ cells (χ^2^ = 4.134, p = 0.04). B) Some animals (33.3%) from the group receiving 25×10^6^ cells were alive at 20 wks of age, when only 21.4% of the group receiving 10×10^6^ cells and 7.7% of the group receiving 50×10^6^ cells survived. G93A mice administered with 25×10^6^ cells survived until 25 wks of age (22 wks of age–25%, 23 wks of age–16.7%, 25 wks of age–8.3%). Media-injected or CsA-injected mice survived no longer than 20 weeks of age.

When MNC hUCB cells at different doses were administered intravenously into G93A mice at 7–8 wks of age, a significant increase in survival between cell transplanted and Media-injected or CsA-injected groups was determined in mice receiving 25×10^6^ cells (χ^2^ = 4.134, p = 0.04, Figure 1, A). Average lifespan of MNC hUCB administered mice was: 10×10^6^–128.3±1.06 days, 25×10^6^–140.5±1.85 days, 50×10^6^–126.3±0.96 days compared to 124.5±1.03 days and 126.9±1.46 days of Media and CsA mice, respectively. Some animals (33.3%) from the group receiving 25×10^6^ cells were alive at 20 weeks of age, when only 21.4% of the group receiving 10×10^6^ cells and 7.7% of the group receiving 50×10^6^ cells survived. Interestingly, G93A mice administered with 25×10^6^ cells survived until 25 wks of age (22 wks of age–25%, 23 wks of age–16.7%, 25 wks of age–8.3%). Media-injected or CsA-injected mice survived no longer than 20 weeks of age (Figure 1, B).

**Figure 2:**
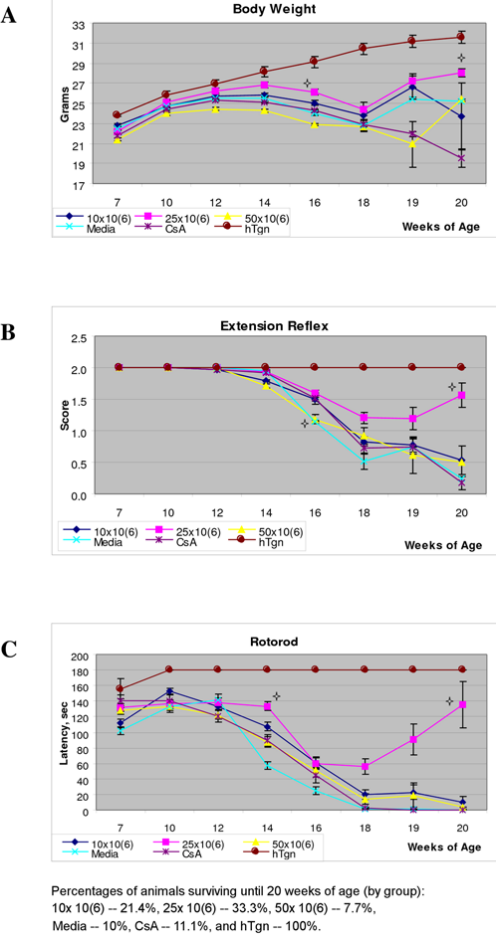
Characteristics of disease progression in G93A mice. A) body weight, B) extension reflex, and C) rotorod test of G93A mice administered with three different cell doses, Media-injected, CsA-injected and control hTgn mice. At 16 wks and 20 wks of age, mice receiving 25×10^6^ cells better maintained their body weight, extended hindlimbs, and stayed longer on rotorod than other cell transplant or control groups. The four pointed stars in A, B, and C indicates significant difference: *in body weight*–16 wks of age: 25×10^6^ cells vs. 50×10^6^ cells (p<0.05); 20 wks of age: 25×10^6^ cells vs. CsA (p<0.05); *in extension reflex*–25×10^6^ cells vs. Media or 50×10^6^ cells (p<0.05); 20 wks of age-25×10^6^ cells vs. CsA or 10×10^6^ cells (p<0. 01); *in rotorod*–14 wks of age: 25×10^6^ cells vs. Media (p<0.05); in 20 wks of age-25×10^6^ cells vs. CsA (p<0.05) or 10×10^6^ cells (p< 0.01).

At 13–14 weeks of age, symptoms of disease progression appeared in the experimental G93A mice. The 14 wks old Media-injected, CsA-injected mice, and mice receiving 50×10^6^ cells (p<0.05) started to lose weight while mice administered with 25×10^6^ and 10×10^6^ cells maintained body weight until 16 wks of age after which, their weight began to slowly decrease. At 16 weeks of age, mice receiving 25×10^6^ cells had significantly (p<0.05) higher body weight compared to mice administered with 50×10^6^ cells. Also, mice with 10×10^6^ cells showed a tendency (p<0.1) to maintain their weight at this time. At 20 weeks of age, mice that had received 25×10^6^ cells better maintained their body weight (28.1±0.41 g) than other cell transplant or control groups and significantly (p<0.05) differed from mice receiving daily CsA (Figure 2, A).

Fourteen weeks of age was a critical time, when Media-injected and CsA-injected animals began showing signs of deterioration in extension reflex (Figure 2, B**)**. At this time, mice which had received 10×10^6^ cells or 50×10^6^ cells showed increasing loss of hindlimb extension vs. control animals. The mice transplanted with 25×10^6^ cells showed more instances of hindlimb extension at 16 wks of age (p<0.05) compared to Media or 50×10^6^ cells groups of mice and demonstrated hindlimb extension until 20 wks of age (Figure 2, B**)**. By 20 wks of age, Media and CsA animals had no extension and exhibited hindlimb paralysis. At the same age, mice administered with 25×10^6^ cells better (p<0.01) extended their hindlimbs vs. mice receiving CsA and 10×10^6^ cells.

In the rotorod test, motor deficit of Media or CsA mice was observed as early as 13 wks of age (Figure 2, C**)**. There were no significant differences between mice receiving 10×10^6^ cells, 50×10^6^ cells, Media, and CsA groups in latency at this time. At 13–14 wks of age, mice administered with 25×10^6^ cells performed best in the rotorod test (14 wks of age: latency-133.3±5.32 sec) compared with another cell treated animals (14 wks of age: 10×10^6^ cells [latency-107.2±5.72 sec], 50×10^6^ cells [latency-87.3±6.61 sec]) and had significantly (p<0.05) longer latency compared to Media mice. Media or CsA mice were not able to perform the rotorod test at 18 wks of age, although mice with 10×10^6^ or 50×10^6^ cell administrations still achieved this performance until 19 wks of age. Animals receiving 25×10^6^ cells stayed on the rotorod with high latency at 20 wks of age, significantly better than mice administered with CsA (p<0.05) or 10×10^6^ cells (p<0.01) (Figure 2, C**)**. These tested mice (33.3% of which still survived at this time) exhibited partial dysfunction of their hindlimbs but could still perform this test.

### Effect of MNC hUCB cell administration on cytokine profile

**Figure 3:**
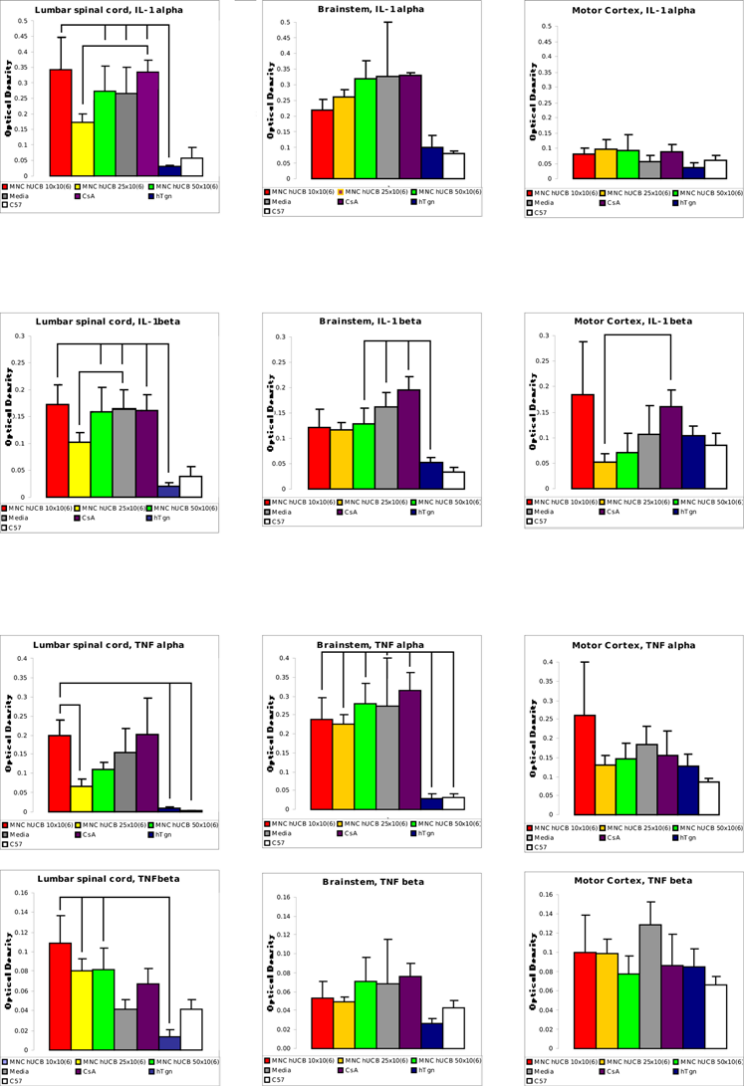
Cytokine profile in the lumbar spinal cord, brainstem, and motor cortex of G93A mice administered with different MNC hUCB cell doses. The RNase protection assay was used to determine the mRNA expression of proinflammatory cytokines IL-1 α, IL-1 β, TNF α, and TNF β in the lumbar spinal cord (left column), brainstem (center column), and motor cortex (right column) of G93A mice administered with different MNC hUCB cell doses. Control groups were Media, CsA, hTgn, and C57BL/6 mice. The mRNA expression presented as the optical density (OD) values obtained from each band normalizing against the OD obtained from the L32, a house-keeping gene, band. Lines indicated significant differences (p<0.001, p<0.01, and p<0.05) between mouse groups (see Results).

To evaluate the effect of administered MNC hUCB cells at different doses on neuroinflammatory processes that occur in ALS, we used the RNase protection assay (RPA) to determine the mRNA expression of proinflammatory cytokines. Our results showed significantly higher expression of IL-1 α mRNA in the lumbar spinal cord of Media (p<0.05), CsA (p<0.05), MNC hUCB 10×10^6^ (p<0.05), and MNC hUCB 50×10^6^ (p<0.05) mouse groups compared with those in control hTgn mice (Figure 3). No differences were observed between mice receiving 10×10^6^ or 50×10^6^ cells and Media- or CsA-injected. However, mice administered with 25×10^6^ cells tended to decreased IL-1 α expression in the lumbar spinal cord (p = 0.1) compared to CsA-injected animals. Although, the expression of IL-1 α mRNA in the brainstem did not significantly differ between cell-treated and non-treated G93A mice, a tendency to decreased expression of this cytokine was observed in mice received 10×10^6^ or 25×10^6^ cells. Interestingly, there were no differences in IL-1 α mRNA expression in motor cortex between cell-treated, non-treated G93A mice and control hTgn or C57BL/6 mice (Figure 3).

Similar to results of IL-1 α mRNA expression in the lumbar spinal cord, IL-1 β mRNA was significantly higher in mice receiving Media (p<0.01), CsA (p<0.05), MNC hUCB 10×10^6^ (p<0.05), and MNC hUCB 50×10^6^ (p<0.01) compared to hTgn mice (Figure 3). The tendency to decreased IL-1 β mRNA in the lumbar spinal cord was found in mice administered with 25×10^6^ MNC hUCB cell dose (p = 0.1) compared with those in Media-injected G93A mice. In the brainstem, significant increase of IL-1 β mRNA expression was detected in Media (p<0.05), CsA (p<0.001), MNC hUCB 50×10^6^ (p<0.05) vs. control hTgn or C57BL/6 mice. There were no differences in IL-1 β mRNA expression between G93A mice treated with different cell doses. However, in motor cortex, mice receiving 25×10^6^ MNC hUCB cells showed significant decrease of IL-1 β mRNA expression (p<0.05) compared to CsA-injected mice.

The expression of TNF α mRNA in the lumbar spinal cord was only significantly higher in mice administered with MNC hUCB 10×10^6^ cells compared to hTgn (p<0.05) or C57BL/6 (p<0.05) mice. Decrease of TNF α mRNA was noticed in MNC hUCB 25×10^6^ (p<0.05) compared with those in MNC hUCB 10×10^6^ mouse group (Figure 3). There were no differences in TNF α mRNA expression in the brainstem between cell-treated and non-treated G93A mice; all showing high cytokine expression vs. control hTgn or C57BL/6 mice: Media (p<0.01), CsA (p<0.001), MNC hUCB 10×10^6^ (p<0.05), MNC hUCB 25×10^6^ (p<0.05), and MNC hUCB 50×10^6^ (p<0.01). A similar effect of TNF α mRNA expression between cell-treated and non-treated mice was detected in the motor cortex, however, no significant differences were found in all groups of G93A mice vs. control hTgn or C57BL/6 mice.

Results on the expression of TNF β mRNA in the lumbar spinal cord showed that only mice receiving 10×10^6^ (p<0.01), 25×10^6^ (p<0.05), and 50×10^6^ (p<0.05) MNC hUCB cells significantly increased this cytokine compared to hTgn control mice (Figure 3). No differences in TNF β mRNA expression were found between mice treated with different cell doses. Interestingly, G93A mice receiving CsA showed similar cytokine expression to C57BL/6 mice. In the brainstem and motor cortex, TNF β mRNA expressions were not significantly different between cell-treated and non-treated G93A or control hTgn and C57BL/6 mice.

**Figure 4:**
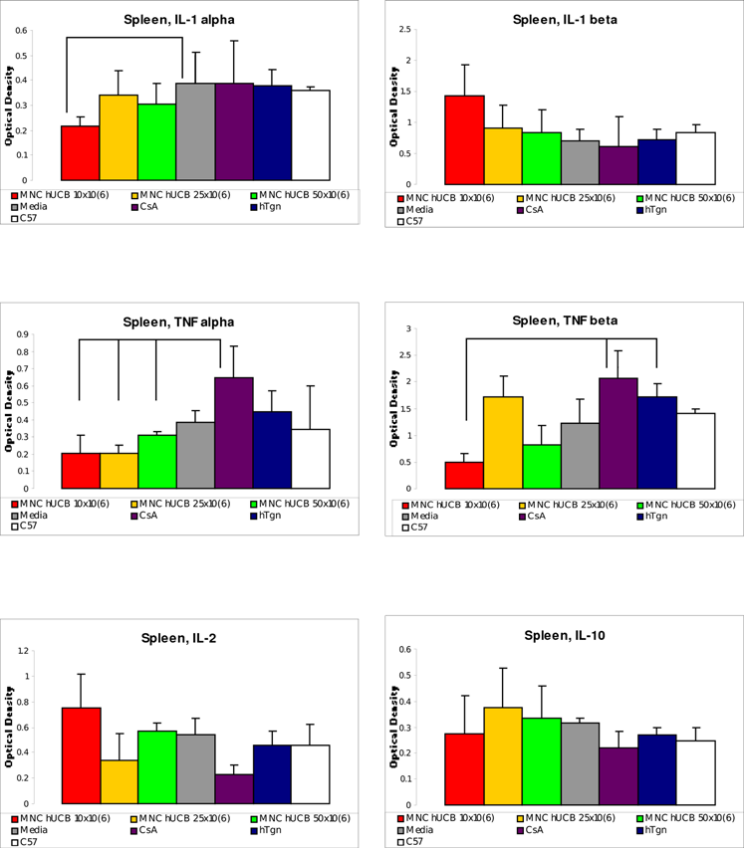
Cytokine profile in the spleen of G93A mice administered with different MNC hUCB cell doses. The RNase protection assay was used to determine the mRNA expression of proinflammatory cytokines (IL-1 α, IL-1 β, TNF α, TNF β, and IL-2) and anti-inflammatory cytokine IL-10 in the spleen of G93A mice administered with different MNC hUCB cell doses. Control groups were Media, CsA, hTgn, and C57BL/6 mice. The mRNA expression presented as the optical density (OD) values obtained from each band normalizing against the OD obtained from the L32, a house-keeping gene, band. Lines indicated significant differences (p<0.001, p<0.01, and p<0.05) between mouse groups (see Results).

The expression of IL-1 α mRNA in the spleen showed a tendency towards decreased levels of this cytokine only in mice receiving 10×10^6^ cells (p<0.1) compared to Media-injected animals (Figure 4). There were no differences in IL-1 β mRNA expression between G93A mice treated with different cell doses and control animals. However, TNF α m RNA expression was significantly decreased in the spleens of mice with 10×10^6^ (p<0.05), 25×10^6^ (p<0.05), and 50×10^6^ (p<0.05) MNC hUCB cell treatments vs. CsA-injected animals. Interestingly, the expression of TNF β mRNA was significantly reduced in mice receiving 10×10^6^ cells compared to either hTgn (p<0.01) or CsA (p<0.05) mice; as a tendency towards a decrease in this cytokine expression was found in mice with 50×10^6^ (p = 0.0571) cell dose vs. CsA-injected mice. Notably, the highest TNF α m RNA and TNF β mRNA expressions were detected in the spleens of mice receiving daily CsA injections (Figure 4). Surprisingly, mice with 25×10^6^ cell treatment also showed high expression of TNF β mRNA similar to mice with CsA. Although, there were no statistical differences in IL-2 mRNA and IL-10 m RNA expressions between G93A mice treated with different cell doses and control animals (Media, CsA, hTgn, C57), mice receiving 25×10^6^ MNC hUCB cells tended towards decreased IL-2 mRNA and increased IL-10 mRNA expressions in the spleen.

**Figure 5:**
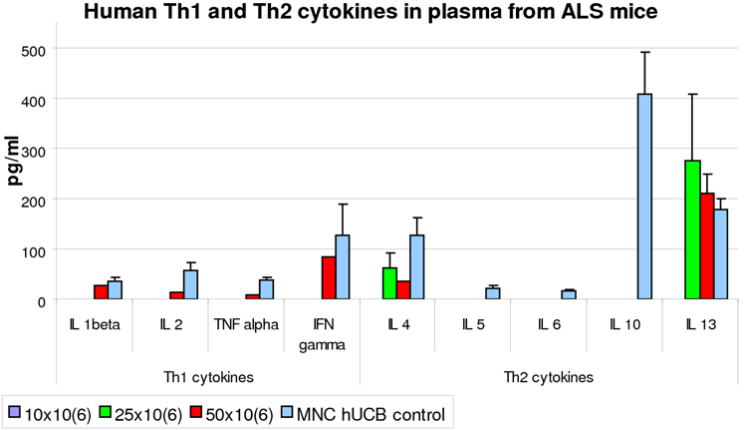
Human Th1/Th2 cytokines in plasma from G93A mice administered with different MNC hUCB cell doses. FAST Quant microspot assay for human Th1/Th2 Cytokines (IL-1 β, TNF α, INF γ, IL-2, IL-4, IL-5, IL-6, IL10, and IL-13) in plasma from G93A mice receiving three different cell doses was performed by Schleicher & Schuell BioScience, Inc. (Keene, NH, USA). Plasma samples from hUCB (n = 10) were used as controls. Quantitative results of cytokines were presented as pg/mL. The 50% of mice receiving 25×10^6^ cells showed evidence of human IL-4 and IL-13 (Th2). In 62.5% of mice with 50×10^6^ cell treatment, all human cytokines were detected except for IL-5, IL-5, and IL-10 (Th2). There were no human cytokines detected in mice treated with 10×10^6^ cell dose. The hUCB plasmas themselves, mostly contained Th2 cytokines (IL-4, IL-10, and IL-13).

To address the question of how transplanted cells provide their beneficial effect, we investigated human Th1 and Th2 cytokines in plasma of G93A mice transplanted with different MNC hUCB cell doses using a microarray assay. Results showed that 50% of mice receiving 25×10^6^ cells showed evidence of human IL-4 and IL-13 (Th2) (Figure 5). In 62.5% of mice with 50×10^6^ cell treatment, all human cytokines were detected except for IL-5, IL-5, and IL-10 (Th2). Interestingly, IL-4 and IL-13 cytokine concentrations in these mice were lower than those in mice receiving 25×10^6^ cells. There were no human cytokines detected in mice treated with 10×10^6^ cell dose. The hUCB plasmas themselves, mostly contained Th2 cytokines (IL-4, IL-10, and IL-13). Although a higher amount of IL-10 was found in hUCB plasma, IFN-gamma (Th1) was also indicated.

### Effect of MNC hUCB cell administration on splenocyte proliferation

**Figure 6:**
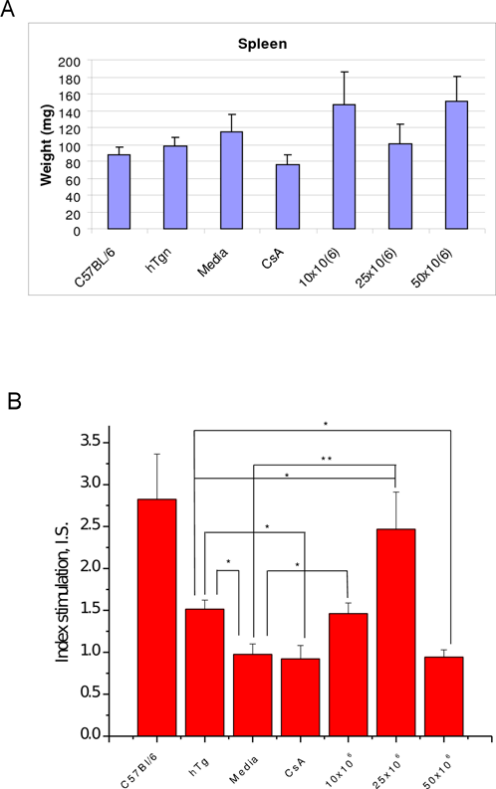
The immune response of splenic cells to a mitogen, phytohemagglutinin (PHA), in G93A mice treated with different doses of MNC hUCB cells. A) The spleen weights in Media mice slightly increase compared to control hTgn or C57BL/6 mice, whereas splenic weight in G93A mice receiving CsA was less. A marked increase of spleen mass was found in mice treated with 10×10^6^ or 50×10^6^ MNC hUCB cell doses, however, high variability in measurements were detected within groups. In mice receiving 25×10^6^ cells, spleen mass was less than other cell treated groups and similar to control animals. B) Index stimulation reflects the ratio between induced PHA proliferation and spontaneous division of splenocytes. There was no splenocyte proliferation found in the Media or CsA injected G93A mice compared to the control hTgn. The mice receiving 50×106 MNC hUCB cells also did not respond to the mitogen. There was a significant increase in splenic cell proliferation of the mice treated with 25×10^6^ (mainly) and 10×10^6^ MNC hUCB cells. The highest amounts of proliferation were found in hUCB and C57BL/6 animals used as controls. *p<0.05 and ** p<0.01.

With evidence that cells colonized the spleen, we investigated the immune responsiveness of splenocytes in treated G93A mice with different MNC hUCB cell doses. The spleen weights in G93A Media mice slightly increase compared to control hTgn or C57BL/6 mice, whereas splenic weight in G93A mice receiving CsA was less (Figure 6, A). A marked increase of spleen mass was found in mice treated with 10×10^6^ or 50×10^6^ MNC hUCB cell doses, however, high variability in measurements were detected within groups. In mice receiving 25×10^6^ cells, spleen mass was less than other cell treated groups and similar to control animals. However, splenocyte proliferation stimulated by PHA was not detected in Media or CsA-treated mice compared to control hTgn or C57BL/6 mice. Surprisingly, reaction to mitogen also was not found in mice receiving 50×10^6^ MNC cells (Figure 6, B). Significant immune response to mitogen (i.e. increased splenic cell proliferation) was found in G93A mice treated with 25×10^6^ (mainly) and 10×10^6^ MNC hUCB cells.

### Effect of MNC hUCB cell administration on hematological response in the peripheral blood

**Figure 7:**
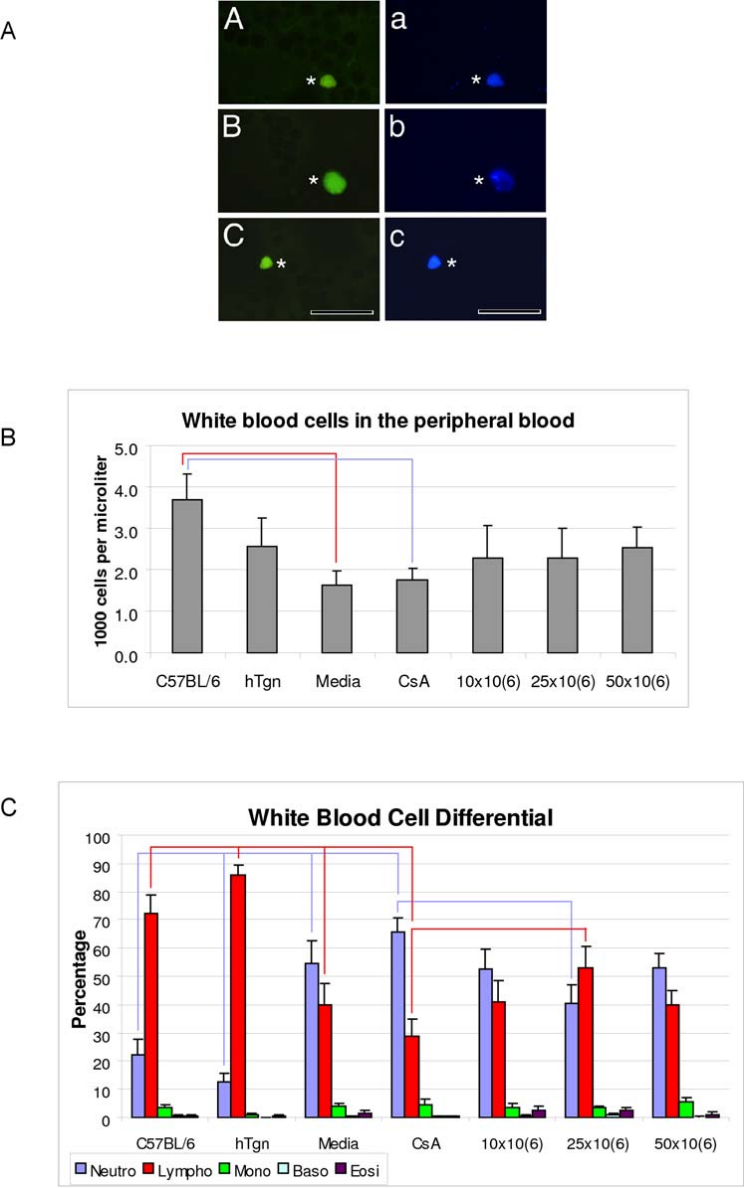
Effect of MNC hUCB cell administration on hematological response in the peripheral blood from G93A mice. A) Many MNC hUCB cells were found in the peripheral blood of mice receiving all three cell doses (A-10×10^6^, B-25×10^6^, C-50×10^6^ cells). Administered human cells were verified by anti-human nuclei staining (green, asterisks). The nuclei in a, b, and c are stained with DAPI, same images as A, B, and C. Scale bar: A-c is 25 µm. B) White blood cell (WBC) counts demonstrated significantly reduced numbers of WBC cells in Media (p<0.05) and CsA-injected (p<0.05) G93A mice compared to C57BL/6 mice. All mice treated with MNC hUCB cells showed slightly increased WBC counts. C) WBC differential analysis showed a low percentage of neutrophils in control hTgn and C57BL/6 mice. A significant increase of neutrophils (almost 4–5 fold) was found in Media (p<0.01) and CsA (p<0.001) vs. hTgn or C57BL/6 mice. Decreased neutrophils in the G93A mice administered with MNC hUCB cells were most significantly pronounced in the 25×10^6^ cell group (p<0.05) compared to CsA-injected mice. The lymphocyte percentage was significantly decreased in Media (p<0.001) and CsA (p<0.001) groups vs. hTgn. Lymphocyte percentages in mice treated with 10×10^6^ and 50×10^6^ cells did not differ from Media mice. In mice receiving 25×10^6^ cells, a significant increase of lymphocytes (p<0.05) vs. CsA-injected mice was determined. Lines indicated significant differences between mouse groups.

For identification of the MNC hUCB cells in the peripheral blood, immunohistochemical staining using human specific antibody (HuNu) was performed. Blood test results showed many transplanted MNC hUCB cells in animals receiving all three cell doses (Figure 7, A). The Complete Blood Count (CBC) in the peripheral blood of G93A mice receiving different MNC hUCB cell doses showed no differences in red blood cell (RBC) count, levels of hemoglobin (Hb) and hematocrit (Htc) compared to Media, CsA, hTgn and C57 BL/6 mice. The RBC average in cell treated mouse groups was: 10×10^6^ cells–8.05, 25×10^6^ cells–8.49, 50×10^6^ cells-8.05 vs. 8.24 (Media), 8.20 (CsA), 7.52 (hTgn), and 7.59×10^6^/µL (C57BL/6) mice. The Hb and Htc amounts were similar in all animal groups averaging 11.58-12-46 g/dL and 32.69–35.63 %, respectively. However, white blood cell (WBC) counts demonstrated significantly reduced numbers of WBC cells in Media (p<0.05) and CsA-injected (p<0.05) G93A mice compared to C57BL/6 mice (Figure 7, B). In contrast, all mice treated with MNC hUCB cells showed slightly increased WBC counts. WBC differential analysis showed a low percentage of neutrophils in control hTgn and C57BL/6 mice averaging 12.60±3.11 and 22.14±5.62, respectively (Figure 7, C). A significant increase of neutrophils (almost 4–5 fold) was found in Media (54.40±8.46, p<0.01) and CsA (65.57±5.09, p<0.001) vs. hTgn or C57BL/6 mice. Decreased neutrophils in the G93A mice administered with MNC hUCB cells were most significantly pronounced in the 25×10^6^ cell group (p<0.05) compared to CsA-injected mice. The lymphocyte percentage was significantly decreased in Media (p<0.001) and CsA (p<0.001) groups compared to those in hTgn. However, lymphocyte percentages in mice treated with 10×10^6^ and 50×10^6^ cells did not differ from Media mice and showed a slight increase when compared to CsA animals. In mice receiving 25×10^6^ cells, a significant increase of lymphocytes (p<0.05) vs. CsA-injected mice was determined (Figure 7, C).

### Effect of MNC hUCB cell administration on microglia in the spinal cord

**Figure 8:**
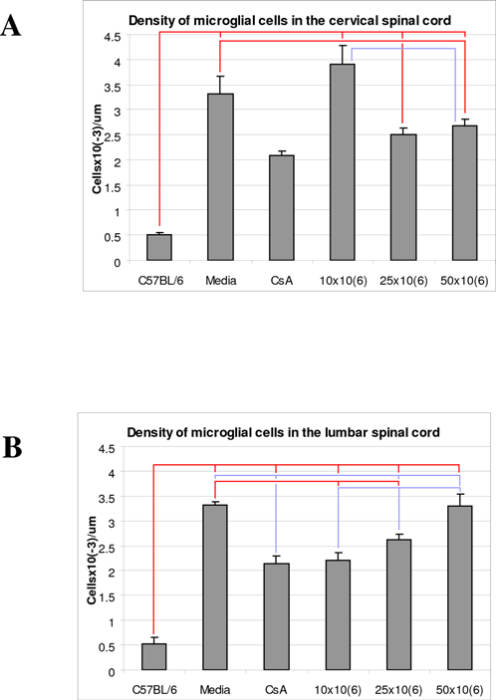
Effect of MNC hUCB cell administration on microglial cell density in the spinal cord of G93A mice. Microglial cell density in the ventral cervical (A) and lumbar (B) horns of all G93A, treated with different cell doses or non-treated (Media, CsA), mice was significantly (p<0.001) increased vs. C57BL/6 animals. Cell density in mice injected with CsA was significantly lower in the cervical spinal cord compared to Media (p<0.01) and 10×10^6^ cells (p<0.001) groups of mice and in the lumbar spinal cord compared to Media (p<0.001), 25×10^6^ cells (p<0.01) and 50×10^6^ cells (p<0.001) groups. A) In the cervical spinal cord, mice receiving 10×10^6^ MNC hUCB cells showed significantly (p<0.001) higher microglial cell density vs. mice with 25×10^6^ and 50×10^6^ cell doses. Animals from these last two groups had microglia of similar appearance and significantly (p<0.01 and p<0.05, respectively) reduced numbers of cells compared to the Media group. B) In the lumbar spinal cord, a significant decrease of microglial cell density was observed in mice receiving 10×10^6^ and 25×10^6^ cells vs. Media mice. Density of microglial cells in mice receiving cell transplants correlated positively with cell dosage. Mice with 10×10^6^ cells had a significantly lower density of microglia than mice administered with 25×10^6^ (p<0.05) and 50×10^6^ (p<0.001) cell doses and cell density was significantly (p<0.05) reduced in mice with 25×10^6^ cells vs. 50×10^6^ cells. Lines indicated significant differences between mouse groups.

Microglial cell density in the ventral cervical and lumbar horns of all G93A, treated with different cell doses or non-treated (Media, CsA), mice was significantly (p<0.001) increased vs. C57BL/6 animals (Figure 8, A, B). However, cell density in mice injected with CsA was significantly lower in the cervical spinal cord compared to Media (p<0.01) and 10×10^6^ cells (p<0.001) groups of mice and in the lumbar spinal cord compared to Media (p<0,001), 25×10^6^ cells (p<0.01) and 50×10^6^ cells (p<0.001) groups. In the cervical spinal cord, mice receiving 10×10^6^ MNC hUCB cells showed significantly (p<0.001) higher microglial cell density vs. mice with 25×10^6^ and 50×10^6^ cell doses (Figure 8, A). Animals from these last two groups had microglia of similar appearance and significantly (p<0.01 and p<0.05, respectively) reduced numbers of cells compared to the Media group. In the lumbar spinal cord, a significant decrease of microglial cell density was observed in mice receiving 10×10^6^ and 25×10^6^ cells vs. Media mice (Figure 8, B). Moreover, density of microglial cells in mice receiving cell transplants correlated positively with cell dosage. Mice with 10×10^6^ cells had a significantly lower density of microglia than mice administered with 25×10^6^ (p<0.05) and 50×10^6^ (p<0.001) cell doses and cell density was significantly (p<0.05) reduced in mice with 25×10^6^ cells vs. 50×10^6^ cells (Figure 8, B).

### Immunohistochemical analysis of administered MNC hUCB cells in vivo

Immunohistochemically, the MNC hUCB cells, identified by human-specific marker (HuNu), were widely distributed in the cervical and lumbar spinal cord of G93A mice treated with different cell doses. The immunophenotype of transplanted cells was verified by double-staining for Nestin.

**Figure 9:**
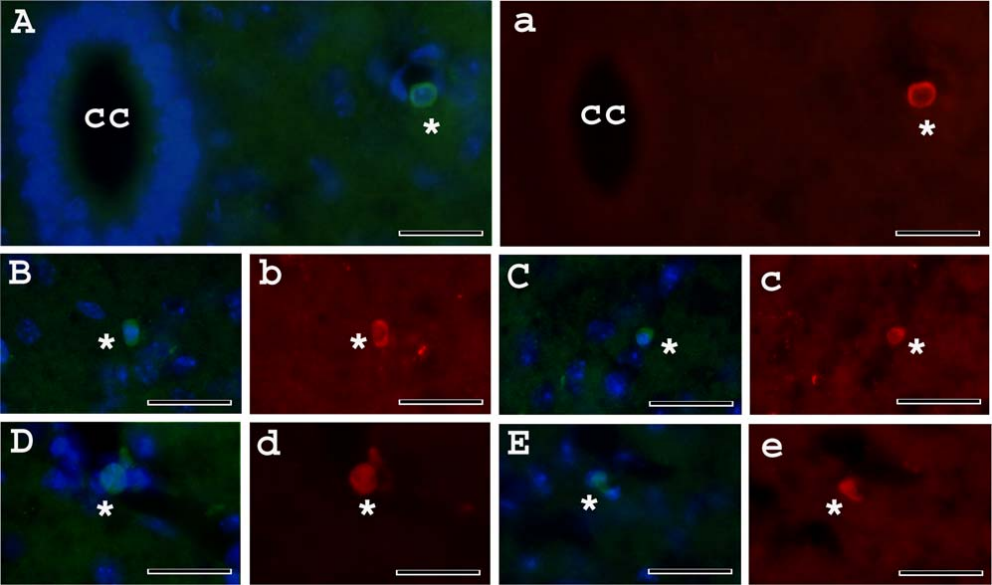
Immunochistochemical staining of MNC hUCB cells in the cervical spinal cord of G93A mice administered with different cell doses. MNC hUCB cells were found in the cervical spinal cord of mice receiving A) 10×10^6^; B), C) 25×10^6^; and D), E) 50×10^6^ cells by anti-human nuclei staining (green, asterisks). Merged images are with DAPI. Some a), b), c), d), and e) MNC hUCB cells were Nestin positive (red, asterisks). Cells in images a, b, c, d, and e are same in images A, B, C, D, E. cc–central canal. Scale bar: A–e is 25 µm.

**Figure 10:**
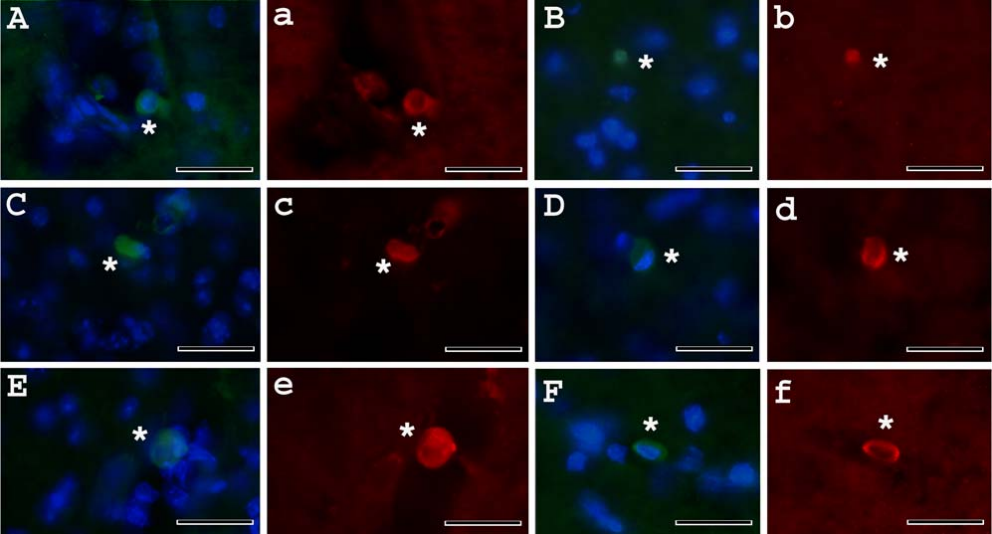
Immunochistochemical staining of MNC hUCB cells in the lumbar spinal cord of G93A mice administered with different cell doses. MNC hUCB cells were found in the lumbar spinal cord of mice receiving A), B) 10×10^6^; C), D) 25×10^6^; and E), F) 50×10^6^ cells by anti-human nuclei staining (green, asterisks). Merged images are with DAPI. Some a), b), c), d), e), f) MNC hUCB cells expressed Nestin (red, asterisks). Cells in images a, b, c, d, e, f are same in images A, B, C, D, E, F. Scale bar: A–e is 25 µm.

In the cervical (Figure 9) and lumbar (Figure 10) spinal cord, targets of motor neuron degeneration in ALS, most cells congregated in, or some distance away from, the blood vessels. Some of the administered cells were found in the parenchyma of the gray matter and expressed Nestin. Notably, administered MNC hUCB cells in the cervical or lumbar spinal cord appeared in several morphologically distinct types with rounded or oval shapes. The HuNu positive MNC hUCB cells were also found in the cervical/lumbar central canal and in white matter of the lumbar spinal cord.

## Discussion

ALS is a multifactor disease characterized by motor neuron degeneration in the brain and spinal cord. Development of an effective treatment is complicated by multiple locations of motor neuron death; cell therapy may be a promising new treatment for ALS. Numerous reports showing the plasticity of hUCB cells in both *in vitro* and *in vivo* opens new approaches for their application in treatment of various disorders including neurodegenerative diseases (see review, [26], [27]). In the present study, we focused on optimization of the MNC hUCB cell dose for treatment of ALS and possible mechanisms of beneficial cell interventions.

The major findings in our study were: 1) administration of the MNC hUCB cells at three different doses (10×10^6^, 25×10^6^, and 50×10^6^ cells) into the systemic circulation of G93A mice showed that that a cell dose of 25×10^6^ cells significantly increased lifespan of mice by 20–25% and delayed disease progression by at least 15%; 2) the most beneficial effect on decreasing pro-inflammatory cytokines in the brain and spinal cord was found in mice with 25×10^6^ cells; 3) human Th2 cytokines were found in plasma of mice receiving 25×10^6^ cells, although prevalent human Th1 cytokines were indicated in mice with 50×10^6^ cells; 4) high response of splenic cells to mitogen (PHA) was indicated in mice receiving 25×10^6^ (mainly) and 10×10^6^ cells; 5) significantly increased lymphocytes and decreased neutrophils in the peripheral blood were found only in animals receiving 25×10^6^ cells; 6) stable reduction in microglia density in both cervical and lumbar spinal cords was noted in mice administered with 25×10^6^ cells; 7) MNC hUCB cells expressing Nestin were found in the cervical and lumbar spinal cords of all mice receiving MNC hUCB cells.

Thus, our results show that the optimal MNC hUCB cell dose for treatment of G93A SOD1 mice is 25×10^6^ cells, which significantly increased lifespan of mice and delayed disease progression. Over thirty three percent of mice from this group were alive at 20 wks of age, when only 21.4% of the group receiving 10×10^6^ cells and 7.7% of the group receiving 50×10^6^ cells still survived. The control (Media-injected or CsA-injected) mice survived no longer than 20 weeks of age. Even this modest percentage (33.3) of surviving G93A mice, “high copy” mice carrying ∼25 copies of mutated gene, which received 25×10^6^ cells, proves the effectiveness of this cell dose. Along with survival, importantly, disease progression was delayed and motor ability retained in these mice. Compared to mice receiving other cell doses and Media-injected or CsA-injected mice, this transplant mouse group showed superior motor function, limited only by a slight decrease in body weight and these mice kept some movement ability until late stage disease. Additionally, a modest positive effect on motor function was observed in mice receiving 10×10^6^ cells.

The first report by Ende et al. [32]) on a possible treatment for ALS with MNC hUCB cells showed that intravenous administration of a high dose (35×10^6^ cells) of MNC hUCB substantially increased the lifespan of mice [32], [33]. The hUCB cells were transplanted into irradiated mice and the authors [32] suggest that these cells may possibly “provide enhanced hematopoietic reconstitution of the irradiated hosts own stem cells.” While the survival data was impressive, the investigators did not examine motor function in these animals or determine the underlying mechanism(s). Presently, our most beneficial cell dose (25×10^6^ cells) is lower than described above and our G93A mice were non-irradiated. Another cell dose (10×10^6^ cells) we investigated had only modest therapeutic effect in G93A mice and results were similar to the effects of low (10^6^ cells) cell administration described in our previous study [31]. There likely is a threshold dosage, that these low doses did not attain necessary to provide significant neuroprotection for motor neurons. Surprisingly, administration of the highest MNC hUCB cell dose (50×10^6^ cells) into G93A mice did not show a curative effect; only 38% of these mice survived at 18 wks of age compared to 71% (10×10^6^ cells) and 83% (25×10^6^ cells) at this age. It is possible that the 50×10^6^ cell dose is high enough to induce immunological “conflict”, similar to the effect seen in graft-versus-host disease. Additionally, a bell-shaped dose response curve balancing neuroprotective and potentially toxic effects might explain the observed effect of the highest cell dose, as is commonly seen in pharmaceutical drug testing.

In ALS, in the brainstem and spinal cord of both patient and animal models, increasingly large numbers of activated microglia and astrocytes, IgG, and T lymphocytes [13], [34]–[36] may be capable of secreting the numerous cytokines playing key roles in the inflammatory reaction. The increase of macrophage-cytokines such as interleukin (IL) 1alpha, IL 1beta and IL 1RA, the pro-inflammatory enzyme cyclooxygenase type 2 (Cox-2) [35], [37], and up-regulation of the tumor necrosis factor-alpha (TNF-alpha) gene [38], [39] makes them likely candidates for inflammatory effectors. Interestingly, T-cell derived cytokines (lymphokines) including IL 2, IL 3, and IL 4 are not elevated in G93A mice even at late stages of disease, which led to the suggestion that “lymphocyte contributions to cytokine expression in ALS are likely minor” [38].

Results of our current study showed that expression of proinflammatory cytokines in the lumbar spinal cord, brainstem and motor cortex was structurally variable in G93A mice of both Media and CsA-injected groups. Significant increases of IL-1 α, IL-1 β, TNF α, and TNF β were mostly detected in the lumbar spinal cord and brainstem of these mice compared to control hTgn or C57BL/6 mice. Cytokine measurements in mice receiving 10×10^6^ and 50×10^6^ MNC hUCB cells were identical with Media or CsA-injected mice. In contrast, a tendency to decreased IL-1 α and IL-1 β mRNA expressions in the lumbar spinal cord was found in mice administered with 25×10^6^ MNC hUCB cells. Importantly, these mice showed a significant decrease of TNF α mRNA in the lumbar spinal cord compared to mice receiving 10×10^6^ MNC hUCB cells. Significantly decreased IL-1 β mRNA expression in motor cortex was also detected in mice with 25×10^6^ MNC hUCB cell dose compared with those in CsA-injected mice.

In the spleen, significant decreases of IL-1 α and TNF β were mostly were found in mice receiving 10×10^6^ MNC hUCB cells and all mice treated with different cell doses significantly reduced expression of TNF α mRNA compared to CsA group. Additionally, although mice treated with 25×10^6^ cells had high variability in IL-2 and IL-10 mRNA expressions in the spleen, they markedly decreased and increased these cytokines respectively.

Thus, the most beneficial effect on decreasing proinflammatory cytokines expression in the brain and spinal cord was noticed in G93A mice administered with 25×10^6^ MNC hUCB cells and this effect was observed mainly through inhibition of IL-1 α and IL-1 β mRNA expressions. Since proinflammatory cytokines may be indirect mediators for glial cells' contribution to motoneuron death, the decrease of these macrophage-cytokines might be due to a reduction of activated microglia and astrocytes by administration of the 25×10^6^ MNC hUCB cells. Also, examination of glutamate concentration will be important to show therapeutic effect of MNC hUCB cells, and will be addressed in a future study. In the periphery (spleen), decreases in proinflammatory cytokines expression (IL-1 α and TNF β) were detected in mice with 10×10^6^ MNC hUCB cell dose and reductions of TNF α mRNA expression were seen in mouse groups with 10×10^6^ and 25×10^6^ cell doses. However, mice receiving 25×10^6^ cells showed a tendency to increased expression of IL-10 mRNA. These results were confirmed by findings of human Th1/Th2 cytokines in plasma of G93A mice treated with different cell doses. The Th2 cytokines were found in mice receiving 25×10^6^ cells, whereas prevalent Th1 cytokines were indicated in mice with 50×10^6^ cells.

Another finding in our present study was significant immune response to mitogen (i.e. increased splenic cell proliferation) found in G93A mice treated with 25×10^6^ (mainly) and 10×10^6^ cells, likely due to immunological tolerance and/or active involvement of grafted MNC hUCB cells in the host immune response. Non-responsiveness of splenocytes to mitogen in Media, CsA, and 50×10^6^ cell treated mice might be due to their dysfunctional lymphocytes, possibly indicating deficient immune response in these mice. In the case of the highest cell dose administration (50×10^6^ cells), immune “conflict” might occur between the high dose of introduced MNC hUCB cells and the host immune system.

Previously we demonstrated severe lymphopenia accompanied by spontaneous autorosette formation in G93A mice at terminal stage of disease [40]. Since transplanted MNC hUCB cells were found in the peripheral blood of animals receiving all three cell doses, white blood cell counts showed a slight increase in these groups compared with non-treated control group mice. However, the white blood cell differential counts demonstrated that significantly increased lymphocytes and decreased neutrophils were found only in animals receiving 25×10^6^ MNC hUCB cells. It is possible that restoration of the lymphoid system in G93A mice by administration of this particular cell dose may have elevated the immune “defense” system and inhibited the inflammatory response.

Supporting this working hypothesis, our results on microglia density in the cervical/lumbar spinal cords showed that the most beneficial cell reduction was observed in mice receiving 25×10^6^ and 50×10^6^ cells doses in the cervical segments, whereas mice with 10×10^6^ and 25×10^6^ cell doses mostly reduced cell density in the lumbar spinal cord. However, stable reduction in appearance of microglia in both cervical and lumbar spinal cords was noted in mice administered with 25×10^6^ cells. Interestingly, animals injected with CsA also showed significant decreases of microglia vs. control Media G93A mice; this indication that the immunosuppressive drug itself might also reduce neuroinflammation needs future investigation.

A necessary step in confirming the utility of any proposed treatment for ALS is analysis of surviving spinal motor neurons. However, the present study examined dosage effects of MNC hUCB cells on lifespan and motor function of ALS mice. Since all animals remained in the study until *end-stage* of disease, typically a result of cumulative motor neuron death, analysis of motor neuron death in our study is likely to be uninformative. A histological analysis of surviving spinal motor neurons seems more appropriately performed on mice euthanized at regular intervals after cell treatment. Such an analysis will be performed in the near future to confirm the effectiveness of our cell therapy.

Thus, results of our pre-clinical study indicate that MNC hUCB cells may have therapeutic potential in the treatment of ALS, providing neuroprotection of motor neurons by inhibiting various immune inflammatory effectors. Although the optimal beneficial cell dose is high, a better approach may be multiple administrations of smaller cell doses, an approach we plan to take for our next study. Additionally, since our study only investigated cells administered into pre-symptomatic G93A mice, future investigations should determine effects of cell transplants performed at different stages of disease.

## Materials and Methods

### Animals

All described procedures were approved by the Institutional Animal Care and Use Committee at USF and conducted in compliance with the *Guide for the Care and Use of Laboratory Animals*. Transgenic male mice B6SJL-TgN (SOD1-G93A) 1GUR (G93A; obtained from Jackson Laboratories, Bar Harbor, MA, USA), over-expressing human SOD1, carrying the Gly93→Ala mutation, were used. The G93A mice randomly received MNC hUCB cells at different doses: 10×10^6^ (n = 14), 25×10^6^ (n = 12) and 50×10^6^ (n = 13) into the jugular vein at 7–8 weeks of age. There were four control groups: G93A Media-injected (n = 10), G93A Cyclosporine A (CsA)-injected (n = 10), C57BL/6 (n = 9) and transgenic mice (BL6/SJL) carrying the normal allele for SOD1 gene (n = 5, hTgn). All mice were maintained on a 12:12 h dark:light cycle (light on at 06:00 hours). Room temperature was 23°C. Food and water were available ad libitum.

### Preparation of MNC hUCB cells and transplantation procedure

Cryopreserved MNC hUCB cells (Saneron CCEL Therapeutics Inc., Tampa, FL) were thawed rapidly at 37°C then transferred slowly with a pipette into a 15-ml centrifuge tube containing Isolyte S, pH 7.4 (Braun/McGaw Pharmaceuticals). The cells were centrifuged (1000 rpm/7 min), the supernatant discarded and the process repeated. After the final wash, viability of cells was assessed using the 0.4% trypan blue dye exclusion method prior to and following transplantation. Transplant cell concentrations were adjusted for each group, respectively: 100,000 cells/µl, 250,000 cells/µl and 500,000 cells/µl.

### Surgery

The MNC hUCB cells were delivered intravenously in mice under anesthesia with Isofluorane (2–5% at 2L O_2_/min) as previously described [31]. Briefly, the jugular vein was exposed and isolated using blunt dissection. The vein was ligated and a small hole made with a 26 gauge needle. A 31 gauge needle attached to a 100 µl Hamilton syringe was placed into the lumen of the vein and sutured in place. The cells (10×10^6^, 25×10^6^, and 50×10^6^) in 100 µl of Media (Isolyte S, pH 7.4) were delivered over 5 min. The Media mouse group received 100 µl of Isolate S, the same volume administered to cell transplant mice. The needle was withdrawn, the suture tightened and the incision closed. All animals, excluding the hTgn and C57BL/6 groups, were immunosuppressed with CsA (10 mg/kg ip per day) during the post-transplantation period.

### Characteristics of disease progression

The evaluation of animal disease progression was previously described [31], [41], [42]. All measures of disease progression were performed blind by independent investigators to avoid subjective bias. Body weight was measured weekly throughout the study. Extension reflex and rotorod test were observed one week prior to transplant, at 12, 14, and 16 weeks of age, and then weekly until 20 weeks of age. After the last behavioral test, lifespan was determined.

#### Extension Reflex

The mouse was suspended by the tail and extension of each hindlimb observed. If the mouse showed normal hindlimb extension, a score of 2 was given. A score of 1 indicated partial hindlimb extension. If no extension was observed, the score was 0.

#### Rotorod

The mouse was placed on a 3.2 cm diameter axle rotating at a speed of 16 rpm (Omnitech Rotoscan). The latency (sec) that the mouse stayed on the rotating axle during a 3 minute period was counted.

### Tissue preparation

The criterion for euthanatization was decreased body weight (more than 10 %) and the inability of mice to move and reach food and/or water due to hindlimb paralysis and muscle atrophy. When progression of disease symptoms reached the point of paralysis, half of the mice from each group receiving 10×10^6^, 25×10^6^, 50×10^6^ MNC hUCB cells, Media, CsA, or C57 BL/6 were sacrificed under deep chloral hydrate (10%) anesthesia and perfused transcardially with 4% paraformaldehyde (PFA) in 0.1 M phosphate buffer (PB, pH 7.2). The cervical and lumbar segments of the spinal cord were removed, post-fixed, and then cryoprotected in 20% sucrose in 0.1 M PB overnight. Coronal sections (30 µm) were cut in a cryostat, thaw-mounted on slides, and stored at 20°C for immunohistochemical analysis. The other half of these mice and all hTgn mice were sacrificed under deep anesthesia, then the brain, spinal cord, and spleen were removed. Weight of the spleen was measured. The brain regions (brainstem, motor cortex) and lumbar spinal cord segment were dissected and frozen in liquid nitrogen and stored at −20°C for cytokine assay. Part of the spleen was cut and also frozen following storage at −20°C. Additionally, blood samples were obtained from all animals. About 300 µl of blood was taken transcardially and collected into blood collection tubes with EDTA (K_3_) (Sherwood Medical, MO). Blood smears were made from snips of the animal tails and then fixed with Methanol for 4–6 min and stored at −20°C. Analyses for Cell Blood Count (CBC) and White Blood Cell (WBC) differential were performed by Antech Diagnostics (NY, USA). Samples of plasma were collected and FAST Quant microspot assay for human Th1/Th2 Cytokines (IL-1 β, TNF α, INF γ, IL-2, IL-4, IL-5, IL-6, IL10, and IL-13) was performed by Schleicher & Schuell BioScience, Inc. (Keene, NH, USA) using manufacturer's protocol. Plasma samples from hUCB (n = 10) were used as controls. Quantitative results of cytokines were presented as picogram per milliliter.

### Ribonuclease extraction and Ribonuclease Protection Assay

Ribonuclease Protection Assay (RPA) was performed as previously described [43]. Briefly, total RNA from homogenized tissues was extracted using Qiagen Rneasy minikit (Qiagen, Valencia, CA) according to the manufacturer̀s instructions. Twenty µg of total RNA from each sample was hybridized with antisense, radiolabeled probes, after which free probe and remaining single-stranded RNA were digested with RNase A/T1. Double-stranded RNase-protected fragments were resolved on 5% denaturing polyacrylamide gels. The probe template used was purchased from Pharmingen (San Diego, CA) and included mouse specific sequences for interleukin-1 α (IL-1 α), interleukin-1 β (IL-1 β), tumor necrosis factor α (TNF α) tumor necrosis factor β (TNF β). Additionally, a custom mouse template for IL-2 and IL-10 was used for the spleen. A positive control transcript was made using a probe specific for the ribosomal protein L32, housekeeping gene, in order to calculate the specific activity and achieve a sufficient excess of probe over target for L32. L32 probe was then added to the probe template before the hybridization reaction started. Yeast transfer RNA and rat mRNA were used as negative and positive controls, respectively. Dried gels were placed on a phosphorimager screen for 16 –20 hours. The phosphorimaging screen was subsequently scanned with a phosphorimager (Molecular Image System GS-363; Bio-Rad, Hercules, CA). The images were processed using Molecular Analyst software (Bio-Rad). The intensity of a band in the computer generated image is directly proportional to the amount of radioactivity within the band. The optical density (OD) values obtained from each band were normalized against the OD obtained from the L32 band in that sample by the following expression: (OD of the sample band / OD of the L32 band X 100).

### Splenic lymphocyte proliferation assay

The proliferation rate of splenic lymphocytes from G93A mice treated with different MNC hUCB cell doses was studied using the CyQUANT Cell Proliferation Assay Kit (Molecular Probes, Invitogen) based on green-fluorescent CyQUANT GR dye, which exhibits strong fluorescent enhancement when bound to cellular nucleic acids. The spleens were aseptically removed from mice (n = 5/group) and disrupted by passage through a sterile plastic strainer. Spleen cell suspensions were prepared in RPMI 1640 and then resuspended in same media containing 10% fetal bovine serum (FBS), 2 mM L-glutamine, 10 µg/ml of gentamicin and 50 µM 2-mercaptoethanol. The cells were counted with a V-cell hematological counter (Beckman Coulter) and plated on a 96 well plate at a density of 2.5×10^4^ cells/well. The phytohemagglutinin (PHA) was then added in 10 µg/ml doses. The culture medium was removed after cultivation for 72 hours and cells were frozen in the plate at −70°C until samples were assayed. After thawing the plates containing the cells at RT, 200 µl of green-fluorescent CyQUANT GR dye/cell lysis buffer was added for 5 min. The fluorescent enhancement was measured with excitation at 485 nm and emission at 530 nm using a Multi-Detection Microplate Reader. Index of stimulation was calculated as the ratio between induced PHA proliferation and spontaneous division of splenocytes.

### Immunohistochemistry

#### Immunohistochemical staining of microglial cells in the spinal cord

Serial sections of the cervical and lumbar spinal cord were rinsed several times in PBS to remove the freezing medium. The tissues were incubated in a solution of 40% Methanol and 30% H_2_O_2_ in PBS for 20 min at RT and then placed in a blocking solution of 10% normal goat serum (NGS) and 0.3% Triton X-100 in PBS for 60 min at RT. Then the tissues were incubated with rabbit polyclonal anti-Iba1 antibody (1∶2000, Wako), 3% NGS, and 0.3% Triton X-100 in PBS overnight at 4°C. After incubation, the slides were rinsed in PBS and incubated with biotinylated goat-anti-rabbit secondary antibody (1∶500, Vector Laboratories), 3% NGS, and 0.3% Triton X-100 in PBS for 60 min at RT. After several rinses in PBS, an avidin-biotin-peroxidase (ABC-Elite kit, Vector) with 3,3-diaminobenzidine (DAB, Pierce) were used to verify microglia/macrophages within the spinal cords. Tissues were then coverslipped with Permount and examined under a microscope.

#### Immunohistochemical staining of MNC hUCB cells in the spinal cord and blood smears

For identification of the MNC hUCB cells in the cervical/lumbar spinal cords and blood smears, staining with the human-specific marker (HuNu) as we described previously [31]. Briefly, the mouse monoclonal antibody (HuNu, 1∶50, Chemicon) was combined with the secondary antibody, monovalent goat anti mouse Fab' fragment conjugated to FITC (1∶200; Jackson Immunoresearch). Prior to applying this antibody cocktail, which had been previously incubated for 60 min at RT, on the slides, M.O.M. immunodetection kit (Vector) was used according to manufacturer's protocol to eliminate the fluorescein background staining. After blocking the tissue with M.O.M. blocking solution and 5% normal human serum (NHS) for 60 min at RT, the antibody complex was applied to the tissues and incubated for 60 min at RT. After a few rinses in PBS, the spinal cord sections were double stained for Nestin (1∶200; Chemicon) overnight at 4°C. Next day, the slides were incubated for 2 hrs with appropriate secondary antibodies conjugated to rhodamine (1∶1200, Alexa, Molecular Probes). The sections were coverslipped with Vectashield with DAPI (Vector Laboratories) and examined under epifluorescence using an Olympus BX60 microscope. For blood smears, the immunostaining procedure only for HuNu, as described above, was used.

### Microglial cell count

Analysis of microglial cell density was performed using a computerized image analysis program (Image-Pro Plus, Media Cybernetics, Inc., Silver Springs, MD). Briefly, measurements of cervical/lumbar ventral horn area were first performed by determining the cross-point of a line passing the central canal perpendicular to the midline. Volume of ventral gray matter was determined below this line in the right and left cervical/lumbar spinal cords in coronal sections (n = 4–5/slide) from each mouse group (n = 3–4/group) at predetermined uniform intervals (90 µm). Number of microglial cells was counted within ventral gray matter of cervical/lumbar spinal cords. Density of microglial cells was determined as cell number per µm. Data is presented as average of cell density of both sides.

### Statistics

Data are presented as means±S.E.M. One-way ANOVA with Student-Newman-Keuls Multiple Comparison test was used. The Kaplan-Meier method was used to determine the difference in survival rate between the groups of G93A mice with or without cell transplants and is reported as a chi-squared value (χ2).
